# Supplementary material for: NCOR1 Sustains Colorectal Cancer Cell Growth and Protects against Cellular Senescence
Source: Cancers (Basel). 2021 Sep 1;13(17):4414. doi: 10.3390/cancers13174414 (PMC8430780; doi:10.3390/cancers13174414)
Supplement: Supplementary file 1 [file cancers-13-04414-s001.zip › Figure S1.pdf]

Figure 4a.

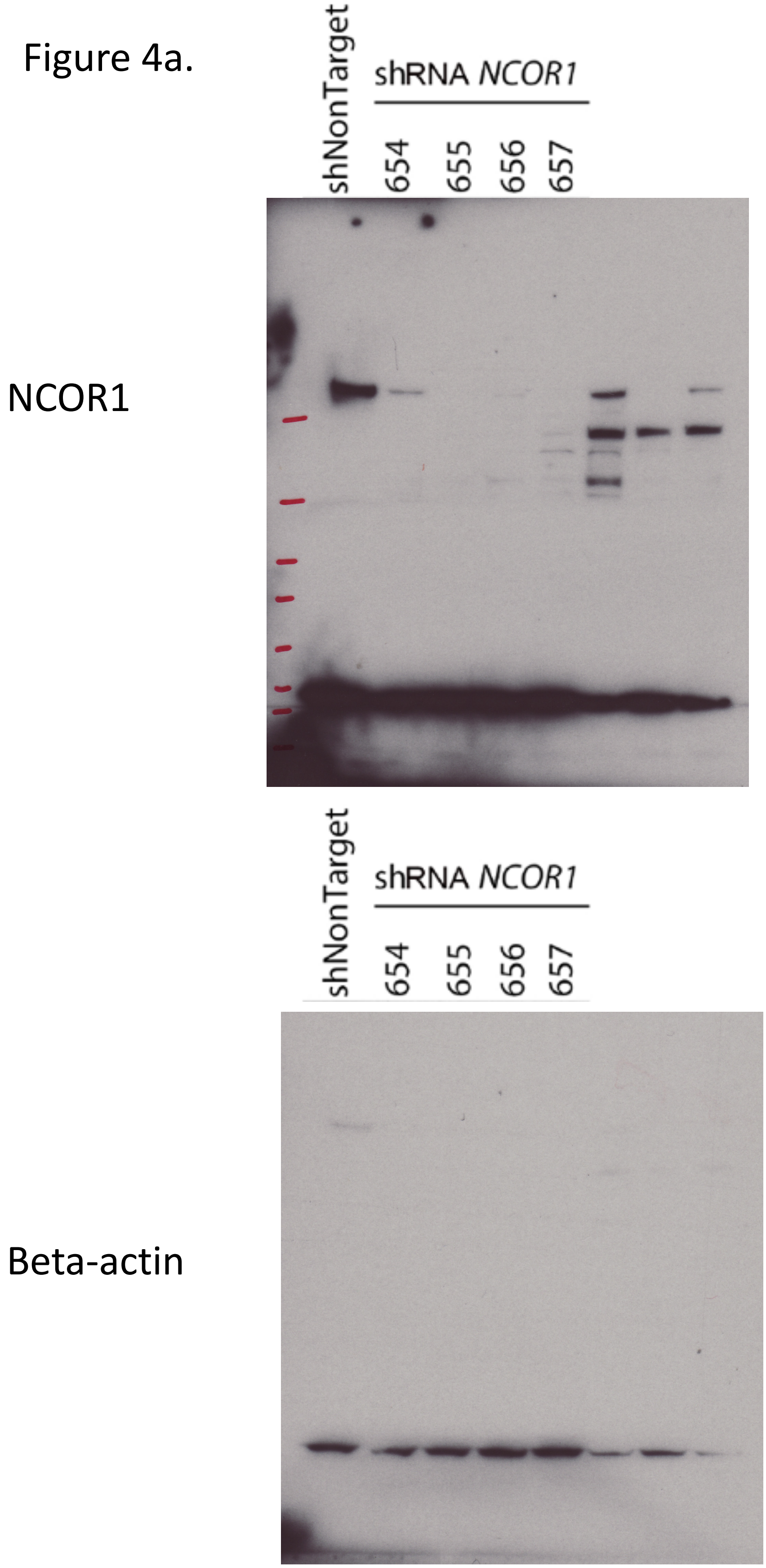
shNonTarget
shRNA *NCOR1*
654
655
656
657
NCOR1
shNonTarget
shRNA *NCOR1*
654
655
656
657
Beta-actin

Figure 4a.

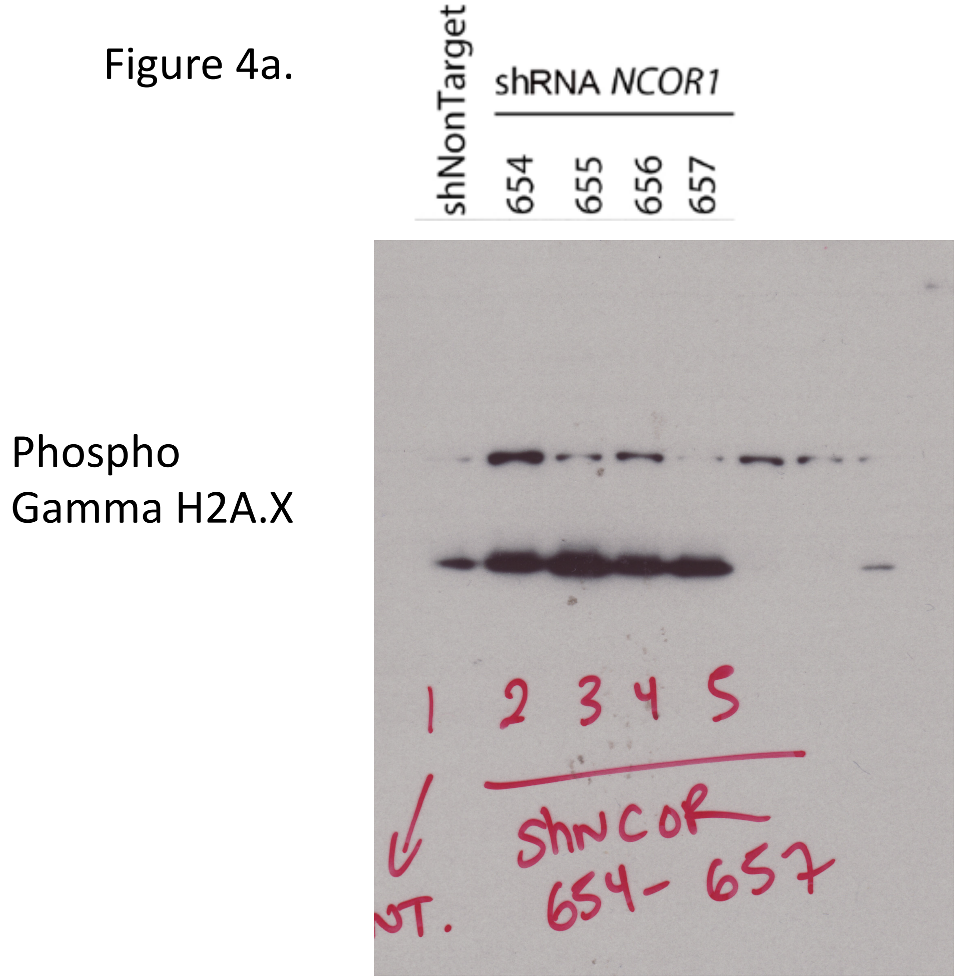

shNonTarget
shRNA NCOR1
654
655
656
657
Phospho Gamma H2A.X
1 2 3 4 5
shNCOR
654- 657
NT.

Figure 9b.

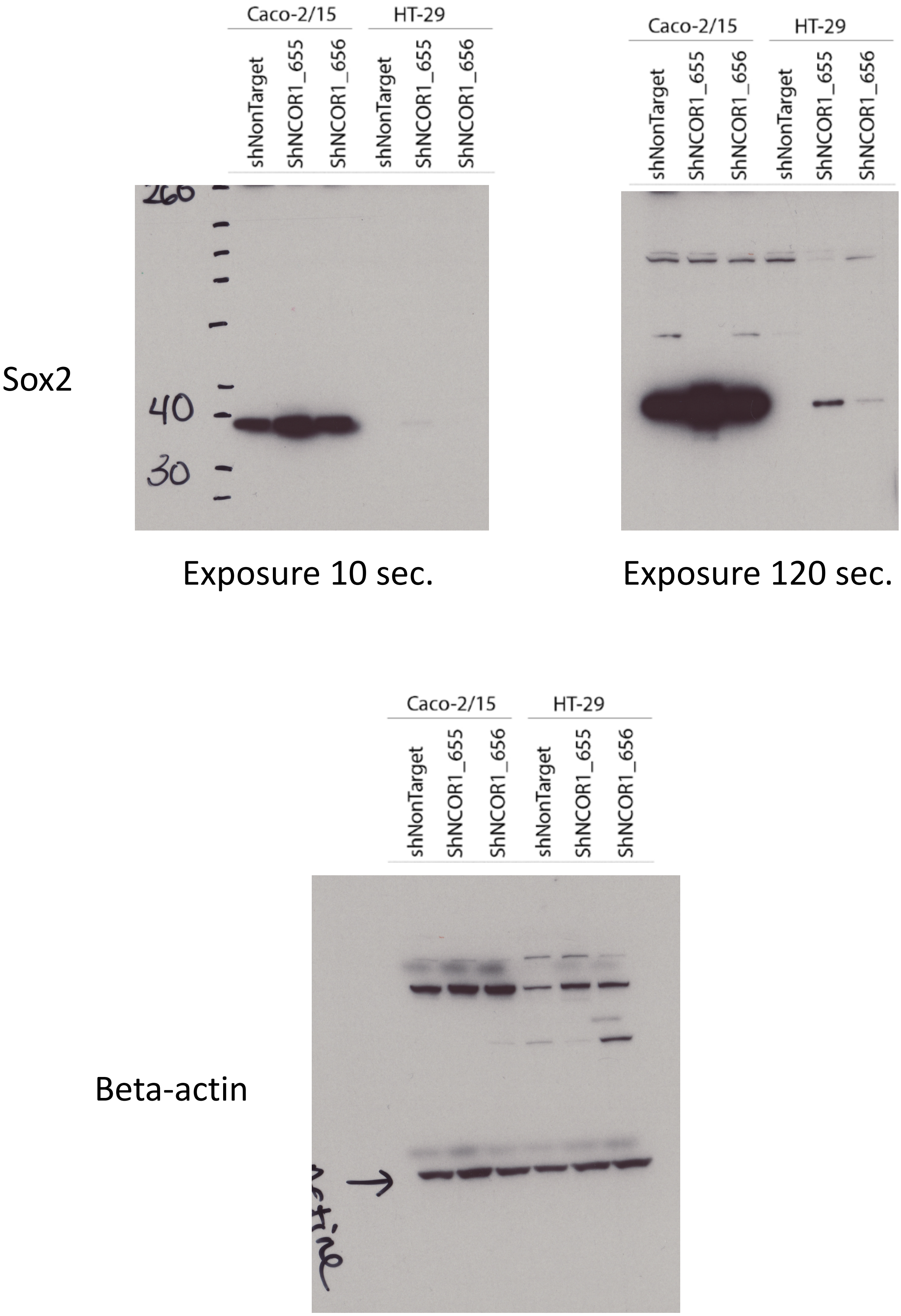
Caco-2/15
HT-29
shNonTarget
ShNCOR1_655
ShNCOR1_656
shNonTarget
ShNCOR1_655
ShNCOR1_656
260
40
30
Sox2
Exposure 10 sec.
Caco-2/15
HT-29
shNonTarget
ShNCOR1_655
ShNCOR1_656
shNonTarget
ShNCOR1_655
ShNCOR1_656
Exposure 120 sec.
Caco-2/15
HT-29
shNonTarget
ShNCOR1_655
ShNCOR1_656
shNonTarget
ShNCOR1_655
ShNCOR1_656
Beta-actin

Figure 3b.

PARP
Caco-2/15

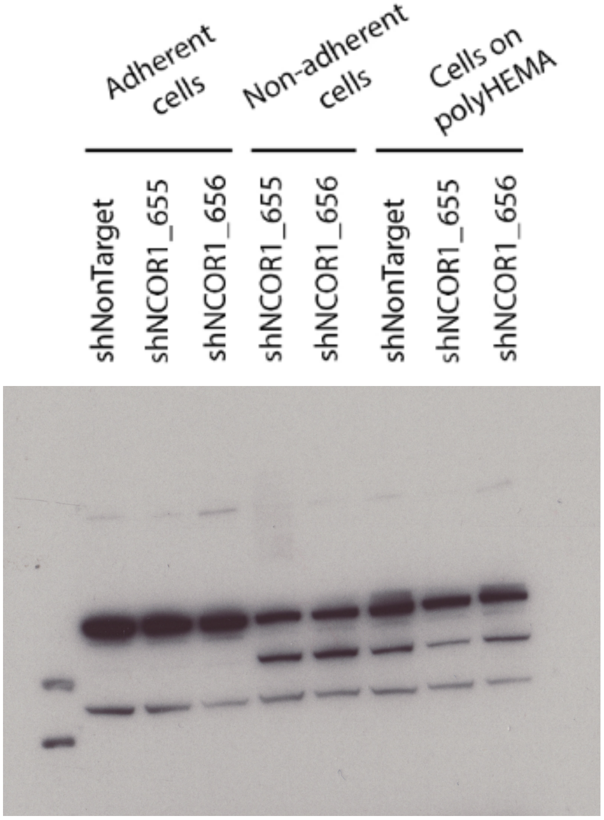

Beta-actin
Caco-2/15

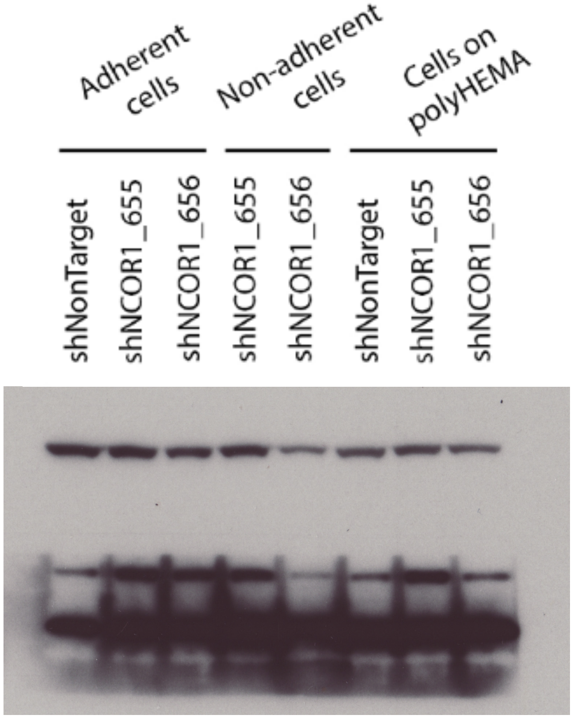

Figure 3b.

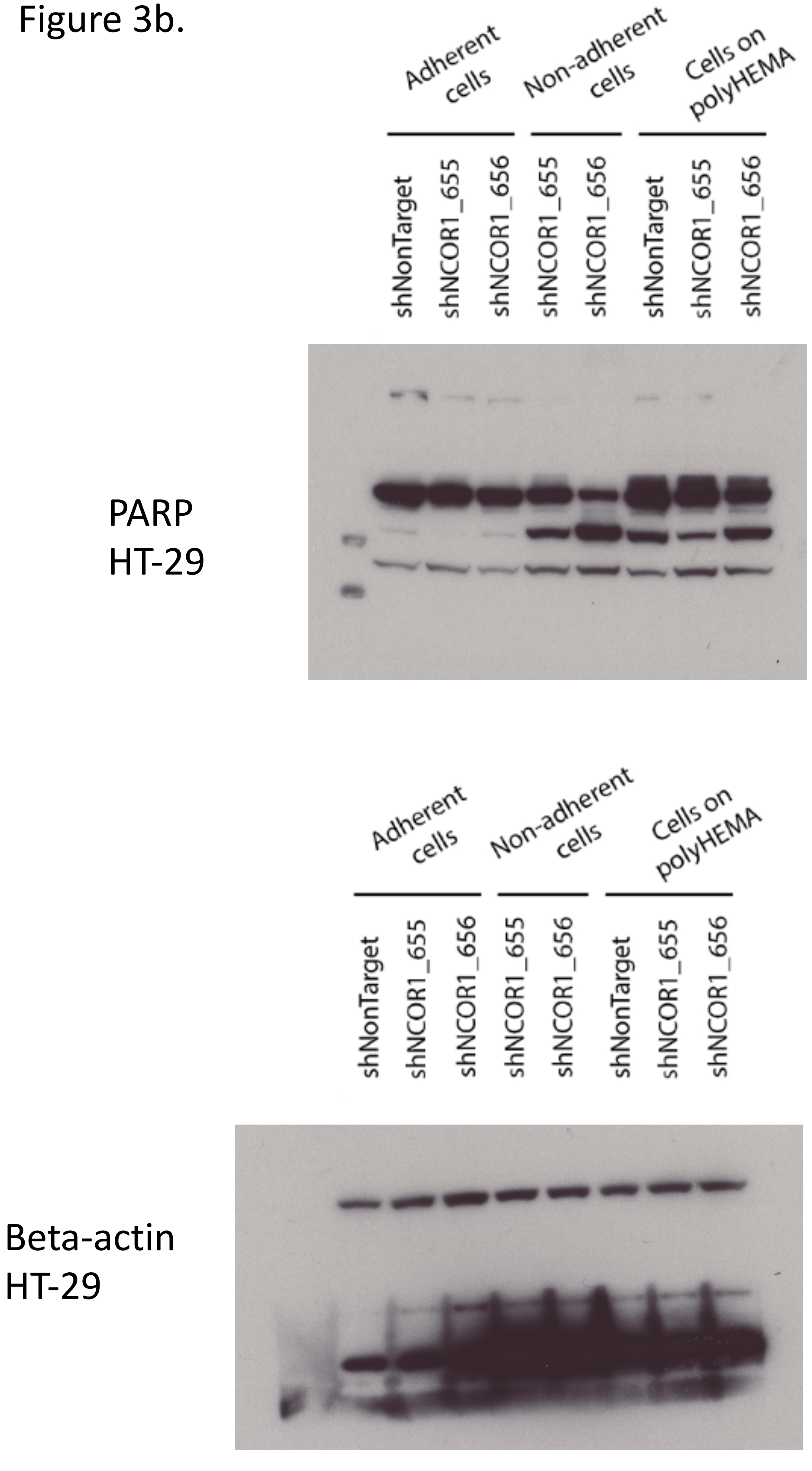
Adherent cells
Non-adherent cells
Cells on polyHEMA
shNonTarget
shNCOR1_655
shNCOR1_656
shNCOR1_655
shNCOR1_656
shNonTarget
shNCOR1_655
shNCOR1_656
PARP
HT-29
Adherent cells
Non-adherent cells
Cells on polyHEMA
shNonTarget
shNCOR1_655
shNCOR1_656
shNCOR1_655
shNCOR1_656
shNonTarget
shNCOR1_655
shNCOR1_656
Beta-actin
HT-29
